# Central importance of emotional and quality-of-life outcomes in the public’s perception of face transplantation

**DOI:** 10.1093/bjs/znab120

**Published:** 2021-05-05

**Authors:** D C Murphy, V Hoyle, D Saleh, J Rees, F Bound Alberti

**Affiliations:** Institute of Genetic Medicine, Newcastle University, Newcastle upon Tyne, UK; Northumbria NHS Foundation Trust, Newcastle upon Tyne, UK; Department of History, University of York, York, UK; Department of Plastic and Reconstructive Surgery, Newcastle upon Tyne Hospitals NHS Foundation Trust, Newcastle upon Tyne, UK; School of Psychology, University of Sunderland, Sunderland, UK; Department of History, University of York, York, UK

## Abstract

**Background:**

Face transplantation is a surgical innovation to manage people with severely interrupted facial function and form. How the public perceive face transplantation and its potential implications for the recipient, donor, and society is unclear. The aim of this study was to understand the public perception of face transplantation, including when it is appropriate, what information is required to feel adequately informed, and which factors influence a person’s willingness to donate their face.

**Methods:**

This was a nationwide survey of participants representative of the GB public. A quantitative analysis was performed. Free-text qualitative responses were coded with thematic content analysis and a narrative analysis was constructed.

**Results:**

The survey included 2122 participants. Face transplantation was considered worth the potential risks if it improved an individual’s quality of life, gave them a ‘normal life’, and/or increased their confidence and social interaction. Respondents were worried about the impact face transplantation might have on donor families, especially recipient families adapting to the identity of the donor. Respondents most concerned about the concept of face transplantation were aged at least 55 years (χ^2^(4) = 38.9, *P* < 0.001), women (χ^2^(1) = 19.8, *P* < 0.001) , and Indian/Asian (χ^2^(4) = 11.9, *P* = 0.016).

**Conclusion:**

The public perceive emotional and psychological outcomes as equally as important as, or more important than, surgical outcomes when determining the appropriateness of face transplantation. Future research should focus on measuring and describing emotional and psychological outcomes after face transplantation.

## Teaser

This article explores public perceptions of face transplants, and highlights the importance of psychosocial as well as surgical outcomes. It shows how important qualitative research is in understanding and evaluating facial transplantation as a form of vascular composite allograft. It also highlights the importance of consulting the public, and facial difference communities, in order to account for lived experience and beliefs about face transplants.

## Introduction

The first face transplantation (FT) was reported in 2005. Subsequently, 46 FTs have been performed globally^1^. As FTs become a viable intervention for severely interrupted facial function and form, the scientific literature is tending to focus on technical and quantitative assessments. Efforts to understand the psychological and quality-of-life impacts are less developed^2–4^. Questions about the appropriateness of FT, and its implications for recipients and donor families remain unresolved.

All transplants have ramifications for recipients and families, but additional complexities relating to identity, communication, and well-being accompany FT^5^^,^^6^. Although psychosocial issues are being raised repeatedly by ethicists, the validity of qualitative outcome measures is unclear, and recipient-centred reporting limited^7–9^.

Engaging the public in FT discussions is critical because willingness to donate is integral to developing a transplant service, and because sociocultural expectations contextualize recipients’ experiences. Factors that define success, psychosocially as well as physically, are required. Consultation with the public is also critical to understanding of the appropriateness of FT as a high-cost intervention in a publicly funded national health service.

The aim of this study was to undertake a nationwide survey to determine public understanding of the circumstances in which FT is appropriate; the most important outcome measures; information needed to feel informed; and factors that influence willingness to donate faces.

## Methods

A cross-sectional survey was distributed to anonymous members of the GB (England, Scotland, and Wales) public using the British market research and data analytics firm, YouGov^©10^. Questions were identified by multidisciplinary discussions between surgical teams, psychologists, qualitative researchers, and patient representatives. Ten priority themes were chosen and all efforts were made to limit order-effects bias. Participants received instructions and a short, textual description of FT, citing potential outcomes and risks, without images.

Using a sampling algorithm that assessed eligible participants against the project’s demographic requirements, on 24 February 2020 YouGov^©^ distributed the survey to participants weighted by demographics, including age, sex, and social class. Ethnicities were represented similarly to those of the UK population, although Caucasians were slightly over-represented^11^.

### Quantitative analysis

Analysis was performed using SPSS® version 25 (IBM, Armonk, New York, USA). Characteristics and proportions were compared using the χ^2^ test, with *P* ≤ 0.050 indicating significance. For non-mutually exclusive questions, the percentage (with 95 per cent confidence interval) based on the total in that demographic group is provided.

### Qualitative analysis

Free-text responses were aggregated and uploaded to NVivo 12™. Thematic content analysis coded data and themes were identified based on meanings, word choice, and context of responses. Codes were interrogated for the frequency of themes (N = X, where N refers to the number of responses), co-determinacy and juxtaposition, and a narrative analysis constructed.

## Results

Some 2112 people responded to the survey (*Table 1*). Respondents were less likely to be concerned than not concerned about FT (χ^2^(1) = 12.0, *P* = 0.001 (*Table 2*). Women were more concerned than men (χ^2^(1) = 19.8, *P <* 0.001). Concern was more often expressed by respondents aged at least 55 years compared with other age groups (χ^2^(4) = 38.9, *P* < 0.001), and this group more often considered FT to be never appropriate (χ^2^(4) = 10.4, *P* = 0.034). Concern was more likely to be expressed by respondents of Indian/Asian ethnicity (χ^2^(4) = 11.9, *P* = 0.016).

**Table 1:** Description of respondents who completed the nationwide survey

	**No. of patients** **(*n* = 2112)**
**Age (years)**	
18–24	230 (10.9)
25–34	354 (16.8)
35–44	373 (17.7)
45–54	332 (15.7)
≥ 55	823 (38.9)
**Sex ratio (M : F)**	1019 : 1093
Ethnicity*	
Caucasian	1968 (93.2)
Indian/Asian	62 (3.0)
Mixed race (white and other)	20 (0.9)
Afro-Caribbean	19 (0.9)
Arab	7 (0.3)
Other	20 (0.9)
**Geographical location**	
North England	517 (24.5)
Midlands	356 (16.9)
East of England	216 (10.2)
London and South East England	554 (26.2)
South West England	189 (8.9)
Wales	97 (4.6)
Scotland	183 (8.7)
**Face difference relationship**	
Personal facial difference	33 (1.5)
Family member with facial difference	60 (2.8)
Friend has facial difference	94 (4.4)
None of above	1830 (86.6)
Prefer not to say	107 (4.7)

Values in parentheses are percentages. *Sixteen respondents declined to state ethnicity.

**Table 2:** Association between respondent characteristics and concerns about face transplantation

	No. of participants	Concerned	Not concerned	Never appropriate
**Total**		801 (46)	946 (54)	155 (7.3)
**Age (years)**				
18–24	230 (10.9)	84 (45)	104 (55)	10 (4.2)
25–34	354 (16.8)	117 (41)	171 (59)	22 (6.3)
35–44	373 (17.7)	108 (37)	181 (63)	23 (6.4)
45–54	332 (15.7)	103 (38)	166 (62)	21 (6.4)
≥55	823 (38.9)	388 (54)	325 (46)	79 (9.4)
**Sex ratio (M : F)**	1019 : 1024	342 : 459	505 : 441	86 : 69
**Ethnicity**				
Caucasian	1968 (93.2)	736 (45)	899 (55)	130 (7.0)
Indian/Asian	62 (3.0)	31 (67)	15 (33)	8 (14.3)
Mixed race (white and other)	20 (0.9)	16 (50)	16 (50)	2 (5.7)
Afro-Caribbean	19 (0.9)	5 (33)	10 (67)	0 (0.0)
Other	27 (1.2)	11 (61)	7 (39)	5 (15.6)
**Face difference relationship**				
Personal facial difference	33 (1.5)	15 (49)	16 (51)	2 (6.2)
Family member with facial difference	60 (2.8)	26 (49)	27 (51)	2 (3.2)
Friend has facial difference	94 (4.4)	40 (47)	45 (53)	2 (2.3)
None of above	1830 (86.6)	697 (45)	844 (55)	133 (7.3)
Prefer not to say	107 (4.7)	28 (58)	20 (42)	16 (14.7)

Values in parentheses are percentages based on respondents who indicated a preference. Participants who responded ‘don’t know’ have been omitted. Percentages of those who said face transplant was never appropriate (never an appropriate intervention under any circumstances) are based on the total number of participants in that subcategory. Abbreviations: F: female; M: male; No.: number

Respondents were asked to describe the circumstances in which FT was an appropriate intervention (*Table S1*). Respondents perceived FT as appropriate when disfigurements were caused by attack, accident or condition (86 (95 per cent c.i. 84-88) per cent), and least appropriate when caused by self-inflicted injury (45 (43-47) per cent), particularly among respondents aged at least 55 years (36 (32-40) per cent), or of Afro-Caribbean (33 (11-55 per cent) or Indian/Asian (19 (6-32) per cent) ethnicity. Respondents with a facial difference agreed that FT was appropriate when psychological health was affected negatively (81 (66-96) per cent), but other groups less commonly held this view.

### Narrative synthesis

Overall, 1610 participants (76 per cent) provided free-text answers in which they centralized emotional and psychological concerns. Potential benefits for recipients were most commonly raised (230 participants), and FT considered worthwhile if it improved quality of life (58), provided a ‘normal life’ (18) or increased confidence (11). Some 109 respondents felt that potential recipients should have autonomy in determining their needs.

Many respondents were concerned about the risk of FT; 112 felt it was too risky, and 110 highlighted dangers of rejection and failure. Negative effects on identity and psychology (33 participants), and the trauma of facial loss for donors and families (39), were described. Many recognized that FT required complex risk–benefit analyses (117 participants) and, where benefits outweighed risks, this reflected positive psychological outcomes.

When asked what further information they required, respondents stressed understanding recipient outcomes (166 participants), rates of success (78), and psychological effects (23). Thirty-seven participants requested testimonies from transplant recipients.

The psychological implications of FT were critical for decisions regarding donation. When considering donation, 107 respondents worried about the impact on their families, especially if recipients resembled donors (68 participants), although this concept is incorrect clinically^12–14^.

Seventy-two participants considered the face too personal to donate; for this reason, 53 would not donate their face, even if they donated other organs. Twenty-seven said this was because the face was visible and personal, unlike internal organs. When asked about donating the face of a loved one, despite 111 expressing a desire to help others, there was significant resistance. Where people would not donate a family member’s face, this was because the idea was upsetting, unbearable or uncomfortable (113 participants).

## Discussion

This large survey of the GB public provides new insights and lay beliefs about FT, and how the public defines success. Emotional and psychological outcomes were equally as important as, or more important than, surgical outcomes. This contradicts existing literature, which largely defines the success of FT in terms of postoperative surgical outcomes.

The importance of recipient autonomy was emphasized by respondents, who believed FT was appropriate if recipients felt that the benefits outweighed the risks. Requests for information about aftercare, outcomes, and prognoses highlights an awareness of a recipient’s longitudinal life experience, especially given the risks of chronic rejection.

It has been argued that individuals cannot give fully informed consent while FT remains experimental; potential recipients are vulnerable, and long-term biopsychosocial outcomes are unknown^15–19^. The present findings suggest that the emotional needs of patients are paramount in public views of FT, and qualitative assessments should investigate the diverse meanings of risk in different contexts.

One area where more clarity is needed is the relationship between cosmetic and functional repair. Respondents overwhelmingly considered FT as intended to improve cosmesis, despite being advised of functional benefits, and requested before-and-after images^20^. Historically, with exceptions, FTs have been reserved for individuals with extreme facial differences and after reconstruction has failed. Most commonly, facial damage results from ballistic injuries, animal attacks, burns, and diseases such as neurofibromatosis. Current indications for FT are more complex than simply cosmesis, and these complexities must form part of organ donor education and risk evaluation for patient and donor families^21–24^.

This cross-sectional survey has measured views in a snapshot rather than over time. Response rates to some questions were low; for example, one-third responded when asked what information would benefit them in the future. Analyses based on low response rates (50 or fewer responses) should be interpreted with caution. The results may be influenced by order-effects bias. Responses are culturally and socially situated in GB. A comparative international survey is needed for generalization.

Defining and measuring emotional outcomes are paramount in the innovation of FT. This will require patient-centred qualitative analysis in combination with clinical data and international collaboration that acknowledges diverse social and cultural contexts. Awareness of social prejudice also needs to be acknowledged, through involvement of patient groups^25^^,^^26^.

## Funding

This research was funded by UKRI, via the Future Leaders Fellowship project AboutFace: The Affective and Cultural History of Face Transplants (MR/S017356/1). The funder had no role in the study design, data collection, analysis or interpretation, in the writing of the report or in the decision to submit it for publication.

## Supplementary Material

znab120_Supplementary_Data

## Notes

Presented to a Head, neck and face virtual meeting organised by the British Association of Plastic, Reconstructive and Aesthetic Surgery on 10th December 2020
