# Supplementary material for: Central importance of emotional and quality-of-life outcomes in the public’s perception of face transplantation
Source: Br J Surg. 2021 May 5;108(10):1145–8. doi: 10.1093/bjs/znab120 (PMC10364884; doi:10.1093/bjs/znab120)
Supplement: znab120_Supplementary_Data [file znab120_supplementary_data.docx]

|  | **Statement number** | | | | | | | |
| --- | --- | --- | --- | --- | --- | --- | --- | --- |
|  | **1** | **2** | **3** | **4** | **5** | **6** | **7** | **8** |
| Total respondents  (N=1582);  %, ±95% CI | 75, ±2·1 | 70, ±2·3 | 58, ±2·4 | 71, ±2·2 | **86**, ±1·7 | 45, ±2·4 | 65, ±2·4 | 56, ±2·4 |
| **Gender**  %, ±95% CI |  |  |  |  |  |  |  |  |
| Male (N=736) | 75, ±3·1 | 69, ±3·4 | 59, ±3·5 | 71, ±3·3 | **88**,  ±2.3 | 44, ±3·6 | 66, ±3·4 | 58, ±3·6 |
| Female (N=846) | 75, ±2·9 | 72, ±3·0 | 57, ±3·3 | 71, ±3·1 | **84**, ±2·5 | 45, ±3·4 | 63, ±3·3 | 54, ±3·4 |
| **Age in years**  %, ±95% CI |  |  |  |  |  |  |  |  |
| 18-24 years (N=183) | **81**, ±5·6 | 70, ±6·6 | 55, ±7·2 | 72, ±6·5 | **80**, ±5·8 | 51, ±7·2 | 59, ±7·1 | 52, ±7·2 |
| 25-34 years (N=265) | 81, ±4·8 | 68, ±5·6 | 59, ±5·9 | 70, ±5·5 | **84**, ±4·4 | 50, ±6·0 | 63, ±5·8 | 58, ±5·9 |
| 35-44 (N=271) | 75, ±5·1 | 69, ±5·5 | 55, ±5·9 | 68, ±5·6 | **84**, ±4·4 | 50, ±6·0 | 67, ±5·6 | 59, ±5·9 |
| 45-54 (N=246) | 77, ±5·3 | 71, ±5·7 | 57, ±6·2 | 74, ±5·5 | **91**, ±3·5 | 50, ±6·2 | 73, ±5·6 | 63, ±6·0 |
| 55+ (N=617) | 70, ±3·6 | 72, ±3·6 | 60, ±3·9 | 70, ±3·6 | **87**, ±2·6 | 36, ±3·8 | 63, ±3·8 | 51, ±3·9 |
| **Relationship with facial difference**  %, ±95% CI |  |  |  |  |  |  |  |  |
| I have a facial difference (N=26) | 75, ±16·4 | **81**, ±15·1 | 58, ±18·8 | 75, ±16·5 | **81**, ±14·9 | 34, ±18·0 | 64, ±18·3 | 50, ±19·0 |
| A member of my family has a facial difference (N=50) | 65, ±13·1 | 62, ±13·4 | 61, ±13·4 | 71, ±12·5 | **85**, ±10·0 | 50, ±13·8 | 68, ±12·9 | 58, ±13·6 |
| A friend of mine has a facial difference (N=84) | 73, ±9·5 | 66, ±10·1 | 58, ±10·5 | 71, ±9·7 | **83**, ±8·0 | 42, ±10·5 | 62, ±10·4 | 56, ±10·6 |
| None of these (N=1394) | 76, ±2·2 | 71, ±2·4 | 58, ±2·6 | 71, ±2·4 | **87**, ±1·8 | 46, ±2·6 | 66, ±2·5 | 56, ±2·6 |
| Prefer not to say (N=38) | 51, ±15·8 | 43, ±15·7 | 32, ±14·8 | 56, ±15·7 | **66**, ±15·0 | 21, ±12·9 | 33, ±14·8 | 35, ±15·1 |
| **Ethnicity**  %, ±95% CI |  |  |  |  |  |  |  |  |
| Caucasian (N=1486) | 75, ±2·2 | 71, ±2·3 | 59, ±2·5 | 71, ±2·3 | **87**, ±1·7 | 45, ±2·5 | 65, ±2·4 | 56, ±2·5 |
| Mixed-race (N=26) | **78**, ±15·9 | **77**, ±16·3 | 51, ±19·3 | 72, ±17·3 | **77**, ±16·1 | 47, ±19·2 | 65, ±18·4 | 58, ±19·0 |
| Indian/Asian (N=36) | 65, ±15·6 | 58, ±16·1 | 38, ±15·9 | 49, ±16·3 | **68**, ±15·2 | 19, ±12·8 | 45, ±16·2 | 46, ±16·3 |
| Afro-caribbean (N=17) | 68, ±22·2 | 61, ±23·3 | 49, ±23·9 | 63, ±23·1 | **72**, ±21·4 | 33, ±22·4 | 56, ±23·7 | 43, ±23·7 |

.

Table S1: Under which circumstances is face transplantation appropriate

Percentage of respondents in agreement with each statement based on their characteristics. Number (N) in first column is the number in the category who agreed with at least one of these statements (excluded those who chose never, don’t know or prefer not to say). The highest agreeing question in each category is marked as bold and the lowest is underlined. For many outcomes, particularly where the number of respondents is large, we can see clear differences because 95% confidence intervals do not overlap. Where subgroups are small, precision is poorer.

The statements are as follows: under which, if any, of the following circumstances would you consider face transplants to be an appropriate treatment for people with severe facial disfigurements?

1. When a person is otherwise unable to smell, eat and talk
2. When a person's psychological health is negatively affected by the appearance of their face
3. When a person finds it extremely difficult to enter social situations (e.g. to have friends and relationships, go shopping in public, etc.)
4. When a person is contemplating suicide because of their condition
5. When a person's disfigurement was caused by attack, accident or medical condition (e.g. following acid burns, animal attack, gunshot, etc.)
6. When a person's disfigurement was caused by a self-inflicted injury (e.g. attempting suicide)
7. When a person was born with their facial difference
8. When a person developed their facial difference at a young age and have lived with it for many years
